# Explaining Variations in Long-term Care Use and Expenditures Under the Public Long-term Care Insurance Systems: A Case Study Comparison of Korea and Japan

**DOI:** 10.34172/ijhpm.2022.6640

**Published:** 2022-11-19

**Authors:** Hongsoo Kim, Nan-He Yoon, Youjin Hahn, Hideki Hashimoto

**Affiliations:** ^1^Graduate School of Public Health Department of Public Health Sciences, Institute of Health and Environment & Institute of Aging, Seoul National University, Seoul, Republic of Korea.; ^2^SOCIUM - Research Center on Inequality and Social Policy, University of Bremen, Bremen, Germany.; ^3^Division of Social Welfare and Health Administration, Wonkwang University, Iksan, Republic of Korea.; ^4^School of Economics, Yonsei University, Seoul, Republic of Korea.; ^5^Department of Health and Social Behavior, The University of Tokyo School of Public Health, Tokyo, Japan.

**Keywords:** Long-term Care System, Performance Assessment, Blinder–Oaxaca Decomposition, Cross-national Analysis, Japan, South Korea

## Abstract

**Background:** Establishing universal coverage of formal long-term care (LTC) services is an urgent policy need for aging populations that requires efficient management of quality and financing. Although current variation in LTC service use between and within countries suggests the potential for improvement by efficient management, this topic remains underexamined. We aimed to identify the sources of variance in LTC use and expenditures through a unique cross-country comparison of Japan and South Korea, which have formal public LTC insurance (LTCI) schemes that are analogous but have unique operational and demographic structures.

**Methods:** Taking administrative regions as the unit of analysis, we assembled data on the LTC utilization rate of people aged ≥65 years, and expenditures per recipient from 2013 to 2015 as the outcome variables. Explanatory variables included demand-related factors, such as regional demographic and economic conditions, and supply characteristics derived from existing public databases. We conducted weighted least squares regression with fixed effects for the pooled data and used Blinder–Oaxaca decomposition to identify sources of outcome variance between the two countries.

**Results:** The average LTC utilization rate was 6.8% in Korea and 18.2% in Japan. Expenditures per recipient were approximately 1.4 times higher in Japan than in Korea. The difference in the utilization rate was mostly explained by between-country differences in supply- and demand-related factors, whereas the difference in expenditures per recipient was largely attributed to unobserved country-specific factors.

**Conclusion:** The current findings suggest that LTC utilization is determined largely by the demographic and functional characteristics of older people, whereas expenditures are more likely affected by institutional factors such as the insurance governance scheme and the policy choice of the target population segment and coverage. The results suggest that strategic choice of LTC institutional schemes is required to ensure financial sustainability to meet changing demands caused by population aging.

## Background

**Key Messages**

** Implications for policy makers**

- Designing universal coverage of long-term care (LTC) for older adults in need is a common policy challenge in societies with population aging.
- Supply and demand factors such as demographic change and provider response are likely to affect the utilization rate of formal LTC; however, policy responses to these challenges are necessarily reactive rather than proactive.
- Institutional factors such as insurance policy design and governance structure have greater effects on utilization cost per service recipient, so should be the main focus of policy design.
- Policy-makers should strategically consider the institutional design of LTC schemes for cost control to efficiently meet increasing demand driven by demographic change.

** Implications for the public**

How to deliver quality long-term care (LTC) for everyone who needs it is a pivotal healthcare policy agenda item in countries facing population aging, and meeting demand with limited financial resources is a challenge. To draw lessons about the efficient management of LTC systems, we examined the cases of Japan and South Korea, which have similarly implemented public LTC insurance (LTCI) systems, but have different levels of population aging and different LTCI system structures. The analysis indicated that the service use rate in the older adult population is likely to be determined by the proportion of the population composed of older adults and their functional limitation severity, which cannot be effectively counteracted by policies. However, expenditures per service recipient are affected more by policy design, and so should be the main target of policy-making. Careful system design and policy decision making are needed to efficiently meet the increasing demand for LTC while retaining the financial sustainability of the system.

Universal coverage of long-term care (LTC) needs is an urgent healthcare policy agenda in countries with rapidly aging populations.^1,2^ With increasing demand for LTC, how to maintain the financial sustainability of public LTC insurance (LTCI) systems in the context of high population aging rates is a common challenge that requires efficient policy management.

 To identify a potential leverage for policy intervention, some cross-national studies have examined variation in LTC use.^3-6^ One previous study used multicountry survey data to compare the extent of and factors associated with formal and informal care use under public LTCI schemes in Germany and the Netherlands.^5^ The findings identified a higher use of formal care in the Netherlands than in Germany that was largely explained by between-country institutional differences, including different eligibility rules and generosity of coverage. However, another study that used descriptive statistics derived from the same data source differently concluded that the effect of institutional factors was multifaceted, and could not be attributed solely to system performance indicators such as the copayment rate and eligibility criteria.^6^ The authors argued that the institutional difference between the two countries was deeply rooted in social preferences for institutional care compared with home care that have been shaped by the culturally and historically unique context of each country about care for older people with needs.^6^

 To further extend our knowledge of LTC policy management, in this study, we conducted a comparative analysis of Korea and Japan as a unique test of the institutional effect on LTC system performance. We compared Korea and Japan for several reasons. The two countries share social norms about care provision for older adults that are rooted in shared Confucianism-based cultures of seniority and family obligation regarding informal care.^7^ Additionally, Korea’s basic public LTC system policy was based on that of Japan.^8^ However, the population of Korea is demographically younger than that of Japan,^1^ and the design of the administrative and operative governance structure of the public LTC systems in the two countries differs.^2,8-10^ An examination of the sources of variance in LTC service utilization and cost in the shared politico-historical contexts of these two countries would help to focus more on the effect of differences in policy governance structures, rather than on differences in the social preferences underlying consumer behaviors.

 More specifically, we investigated cross-country differences in the LTC utilization rate (ie, consumer choice under the eligibility certification) and expenditures per recipient (ie, service intensity under the payment scheme) to identify the contributions of supply and demand factors, such as the demographic characteristics and care needs of older adult recipients and regional healthcare resources. We regarded the utilization rate as an indicator of “coverage” and the expenditure per recipient as an indicator of “intensity,” in accordance with previous discussions of LTC financing.^11,12^ We hypothesized that coverage is influenced mainly by demand factors related to population aging and subsequent needs for LTC. Although the coverage should also be related to policy generosity and supply factors, these variables are likely to be a response to increased demand, as seen in a politico-historical analysis of the public LTC systems in Germany, Japan, and Korea.^2,13^ Instead, we expected that the intensity is more likely to be affected by policy discretion and public payer’s concerns about financial sustainability. To draw out policy implications related to the efficient management of social LTCI schemes, we examined how much variance in coverage and intensity was left unexplained by demand and supply factors, assuming that the unexplained portion would reflect the significance of country-specific institutional factors such as the governance scheme and policy design of the LTC systems. Our aim was to draw generalizable lessons about resource allocation between coverage and intensity that would assist policy-makers in other countries with aging populations to efficiently plan future LTC institutional policies.

## Methods

 In this study, we compared LTC systems in Japan and Korea between 2013 and 2015, when the two countries had similar benefit schemes (as explained below). After 2016, Japan’s insurance scheme no longer covered home- and community-based care for people with mild disability. This precluded comparisons between and within countries after 2016.

###  Institutional Background in Brief

 This section provides a brief description of the LTC systems in the two countries during the studied period. A detailed description is provided in Supplementary file 1, and in previous literature.^2,8-10^

 Japan established its social insurance-based system in 2000 with mandated premium contributions from individuals aged 40 years or older. The aim of the system was to provide formal LTC services for individuals aged 65 years or older and those with designated disabilities and approved as eligible according to the national standardized eligibility criteria. Korea adopted a similar scheme by implementing an LTCI system in 2008; however, in the Korean system, all households pay premium contributions to permit wider intergenerational transfer.^9^

 After being approved as eligible, beneficiaries in both countries are allowed to choose the type of service they will receive, within a monthly limit. In Japan, certified service coordinators help beneficiaries choose a service, but such support is not officially available in Korea. The copayment rate is approximately 10% in Japan. In Korea, it is 20% for institutional-care users and 15% for home- and community-based service users.

 When Japan implemented the LTCI system in 2000, the percentage of the population aged 65 years or older was 17.3%. In contrast, when Korea implemented its LTCI system in 2008, the percentage of the population in the same age range was approximately 10%. To meet the wide range of demands for LTC, Japan’s LTCI emphasized home- and community-based care, whereas the Korean LTCI focused more on supporting individuals who had a substantial need for institutional care and limited informal care resources.^2,8^

 Another difference between the two systems is the insurer scheme.^8^ Japan uses a multipayer system with more than 2500 local municipal government insurers, whereas Korea has adopted a single-payer system (the National Health Insurance Service; NHIS).

 The Japanese decentralized system obtains 50% of its funding from local premium revenue, 25% from tax transfers from the central government, and 25% from tax transfers from local (prefecture and municipal) governments. The Korean system is 80% funded by premium revenue collected by the single government payer, or NHIS; the remaining 20% is covered by tax transfers from the central and local governments.

 The LTCI systems in both countries rely heavily on the private sector for service delivery, which is paid for on a fee-for-service basis under nationally standardized fee schedules. In Japan, most of the relevant private sector actors are non-profit organizations, whereas providers with private ownership are more dominant in Korea.

###  Data Sources

 We compiled comparable and complete regional-level data from Japan and Korea for the years between 2013 and 2015. In the Organisation for Economic Co-operation and Development (OECD) Regional Well-being initiative,^14^ prefectures in Japan (*N* = 47) and provinces in Korea (*N* = 17) are used as comparable regional units of analysis. We excluded Sejong city in Korea, which was newly established as an administrative capital in 2012, because of its unique function as a specialized government city. We also excluded the Japanese prefectures of Osaka, Kyoto, Kagawa, and Nagasaki owing to the unavailability of full information regarding LTC service recipients. Therefore, the present analysis included a complete dataset of 59 regional units (43 for Japan, 16 for Korea) for 3 years, or 177 observations.

 For Korea, the *Long-term Care Insurance Statistics Book*, released annually by the NHIS, is the major source of data on LTC beneficiaries, expenditures, and resources, including institutions and the workforce.^15^ We also extracted data from the National Health Insurance Statistical Yearbooks and regional statistics in Korea.^16^ For Japan, we used data collected from four LTC-related data sources: *Statistics of Long-term Care Benefit Expenditure*,^17^ *Survey of Medical Institutions*,^18^ *Statistics of Physicians, Dentists, and Pharmacists*,^19^ and *Local Finance Statistics Annual Report*.^20^ For both countries, regional-level population and income data were obtained from the OECD regional database.^14^ The definitions of data items and data-collection profiles were rigorously and repeatedly checked by the authors in both countries to maximize data comparability while maintaining relevance for the country-specific policy context.

###  Outcome Variables

 The outcome variables of interest in each region were the LTC utilization rate (defined as the number of LTCI beneficiaries with actual formal service use per older adult aged 65 years or older in the population) and LTC expenditures per service recipient. We counted only the utilizations and expenditures of LTC beneficiaries aged 65 years or older in both countries to maintain the comparability of age structure across the datasets. We used the logged values of LTC expenditures in our analysis because this variable was positively skewed.

 For the exchange rates, we used 101.303 JPY/USD (year 2013), 103.052 JPY/USD (year 2014), 103.469 JPY/USD (year 2015), and 869.081 KW/USD (year 2013), 871.878 KW/USD (year 2014), and 857.483 KW/USD (year 2015).^21^

###  Explanatory Variables

 The demand-side elements we investigated comprised predisposing, enabling, and need factors, following a previous study.^3^ As a predisposing factor, we included the proportion of the total population in a region made up by women aged 65 years or older, as the prevalence of morbidity and disability requiring LTC differ systematically by gender.^22^ Because age is also an important predisposing factor, we tried to include the population proportion of individuals aged 80 years or over; however, the regression-based analysis showed a high variance inflation factor (>10). This indicated the presence of substantial collinearity with the need-related variables (defined below) and supply-related variables. Therefore, we decided not to include regional age structure; instead, the outcome variables were expressed per older adult in the population, to take account of population age structure.

 The enabling factors were the household’s purchasing capacity and the local government’s capacity to pay for welfare. Data on household net adjusted disposable income per capita were obtained from OECD regional statistics in tens of thousands of USD. We also calculated the welfare ratio as the percentage of each region’s total budget spent on social welfare expenditures. The needs factors were the fraction of LTCI-eligible older adults rated at the most or the second-most severe eligibility level (ie, the least able) and the overall death rate among older adults in a region.

 Finally, the supply factors were the number of doctors per 100 people; the number of hospital beds per 100 people; the number of LTC institutions per 1000 older adults; the number of care staff (ie, the nursing workforce), including nurses, nursing aides, and personal carers per 1000 older adults; and the proportion of all LTC institutions that were government owned.

###  Statistical Analysis 

 First, we used linear regression models to examine factors that explained the LTC utilization rate and LTC expenditures in each country. We weighted the sample by the proportion of the population in each region made up of older adults and estimated the model using weighted least squares. To account for multiple observations per region/prefecture over the years, sandwich estimation of standard errors clustered by region/prefecture was used.

 Next, we decomposed the outcome gap between Korea and Japan using the decomposition technique proposed by Oaxaca^23^ and Blinder^24^ to determine whether a between-country gap is caused by differences in the means of the covariates reflecting the effect of between-country differences in the distributions of the observed explanatory variables, or by differences in the coefficients unexplained by the observed variables, which presumably reflects the difference attributable to unmeasured institutional differences between the two countries’ systems. Supplementary file 2 provides more details about the analytic methods.

## Results

###  Descriptive Statistics

Table 1 reports summary statistics by country. On average, 6.8% of people in Korea aged 65 years or older were LTC recipients, compared with 18.2% of their counterparts in Japan during the observed years. Average LTC expenditures per recipient were approximately 1.4 times higher in Japan than in Korea.

**Table 1:** Descriptive Statistics

	**Korea** **Mean (SD)**	**Japan** **Mean (SD)**	**Diff. (J−K)**	***P*** ** Value of Difference**
Outcomes				
LTC utilization rate^a^	0.068 (0.010)	0.182 (0.035)	0.113	<.001
Logged LTC expenditure (per beneficiary)^b^	9.294 (0.069)	9.604 (0.184)	0.312	<.001
LTC expenditure per beneficiary^c^	10.880 (0.774)	15.100 (3.197)	4.220	<.001
**Explanatory variables**				
Demand side (predisposing)				
Proportion women aged ≥65 years	0.588 (0.013)	0.574 (0.015)	−0.014	<.001
Demand side (enabling)				
Disposable income^c^	1.611 (0.144)	1.891 (0.187)	0.280	<.001
Welfare expenditure ratio^d^	0.281 (0.078)	0.144 (0.039)	−0.137	<.001
Demand side (need)				
Proportion with severe eligibility levels^e^	0.253 (0.044)	0.312 (0.021)	0.059	<.001
Mortality rate for those aged ≥65 years	0.033 (0.003)	0.036 (0.003)	0.003	<.001
Supply side				
Physicians per 100 population	0.240 (0.057)	0.242 (0.039)	0.002	.917
Hospital beds per 100 population	1.436 (0.457)	1.423 (0.365)	−0.013	.916
LTC institutions per 1000 aged ≥65 years	2.633 (0.475)	6.516 (1.198)	3.883	<.001
Nursing workforce per 1000 aged ≥65 years	44.390 (9.833)	52.848 (6.922)	8.457	.001
Proportion of LTC institutions that are government owned	0.011 (0.011)	0.004 (0.005)	−0.006	.041

Abbreviations: LTC, long-term care; SD, standard deviation. Note: Samples are weighted by the proportion of the population in the region made up of older adults. ^a^The number of LTCI beneficiaries with actual LTC use per adult aged 65 years or older. ^b^LTC expenditure per beneficiary with actual LTC use (in thousands of USD). ^c^Household net adjusted disposable income per capita (in tens of thousands of USD). ^d^Social welfare expenditure as a percentage of each region’s total budget. ^e^The proportion of LTCI-eligible older adults rated at the most or the second-most severe eligibility level.

 In terms of demand-side factors, the percentage of the population made up of older women was higher in Korea (58.8%) than in Japan (57.4%). Disposable income per capita was higher in Japan (approximately US$ 18 910) than in Korea (US$ 16 110). The welfare expenditure ratio in local governments in Japan (14.4%) was about half that in Korea (28.1%), which suggests that the welfare program compensated for differences in purchasing capacity among households more in Korea than in Japan. The regional mortality rate and proportion of older people with a severe need for LTC, reflecting needs level, were both significantly higher in Japan than in Korea.

 Supply-side factors related to medical care were similar in Japan and Korea; the cross-country differences were more remarkable in supply-side factors related to LTC provision. The average numbers of LTC institutions (Japan: 6.5 vs. Korea: 2.6) and LTC care workers (Japan: 52.8 vs. Korea: 44.4) per 1000 older adults in a region were higher in Japan than in Korea. The proportion of LTC institutions in a region that were government owned was quite low in both countries.

###  Determinants of Long-term Care Use and Expenditures

####  Long-term Care Utilization Rate

Table 2 shows the results of the regression analysis predicting the LTC utilization rate by country and in the pooled data.

**Table 2:** Regression Results Predicting LTC Utilization Rate by Country and in the Pooled Data

	**LTC Utilization Rate**^a^		
	**Korea**	**Japan**	**Pooled**
*Demand side*			
Proportion women aged ≥65 years	−0.081	−0.402	−0.333
	[−0.234, 0.071]	[−1.339, 0.535]	[−0.921, 0.256]
Disposable income^b^	−0.015	−0.022	−0.022
	[−0.033, 0.003]	[−0.083, 0.038]	[−0.074, 0.030]
Welfare expenditure ratio^c^	−0.126	−0.202	−0.177
	[−0.154, −0.099]	[−0.381, −0.023]	[−0.317, −0.038]
Proportion with severe eligibility levels^d^	−0.018	0.353	0.208
	[−0.064, 0.027]	[−0.023, 0.730]	[−0.037, 0.452]
Mortality rate for those aged ≥65 years	0.411	1.252	1.499
	[−0.087, 0.909]	[−3.085, 5.589]	[−1.492,4.490]
*Supply side*			
Physicians per 100 population	0.035	0.021	0.046
	[0.000, 0.071]	[−0.247, 0.290]	[−0.120, 0.212]
Hospital beds per 100 population	−0.007	0.012	0.011
	[−0.013, −0.001]	[−0.022, 0.045]	[−0.013, 0.034]
LTC institutions per 1000 population aged ≥65 years	0.007	0.016	0.015
	[0.002, 0.011]	[0.008, 0.024]	[0.008, 0.021]
Nursing workforce per 1000 population aged ≥65 years	0.001	0.001	0.001
	[0.001, 0.001]	[−0.001, 0.002]	[−0.000, 0.002]
Proportion of LTC institutions that are government owned	0.045	0.791	0.247
	[−0.024, 0.114]	[−0.616, 2.198]	[−0.458, 0.951]
*Year*			
2014	0.006	0.003	0.004
	[0.005, 0.008]	[−0.003, 0.008]	[0.000, 0.008]
2015	0.009	−0.005	−0.002
	[0.007, 0.011]	[−0.014, 0.005]	[−0.008, 0.005]
Japan dummy			0.012
			[−0.036, 0.061]
Constant	0.113	0.166	0.147
	[0.000, 0.226]	[−0.299, 0.631]	[−0.213, 0.507]

Abbreviation: LTC, long-term care. Note: Samples are weighted by the proportion of the population in the region made up of older adults. 95% confidence intervals in brackets. ^a^The number of LTCI beneficiaries with actual LTC use per adult aged 65 years or older. ^b^Household net adjusted disposable income per capita (in tens of thousands of USD). ^c^Social welfare expenditure as a percentage of each region’s total budget. ^d^The proportion of LTCI-eligible older adults rated at the most or second-most severe eligibility level.

 In terms of demand-side factors, the utilization rate was lower in regions with a higher proportion of women aged 65 years or over, with higher disposable income, with higher welfare spending and with higher mortality among individuals aged 65 years or over in both countries. The proportion of severe eligibility levels was positively associated with higher utilization rates in Japan, whereas this association was slightly negative in Korea.

 Regarding supply-side determinants, the number of physicians per 100 population, LTC institution supply, and nursing workforce per 1000 population were positively associated with LTC use in both countries. In Korea, the number of hospital beds per 100 population was negatively associated with LTC use, whereas this association was positive in Japan. Because LTC services and medical services for chronic conditions often overlap, it is not unusual for older adults with LTC needs to obtain treatment for chronic conditions from medical facilities instead of LTC in Korea^3^ and Japan.^20^ Because the Korean system somewhat priotises people with high care needs, who often have comorbid conditions requiring medical attention, the negative association between hospital bed availability and LTC utilization because of this kind of substitution of care may be particularly apparent in Korea.

####  Long-term CareExpenditures

Table 3 shows the regression results for predictors of LTC expenditures per service recipient (log-transformed). Compared with the findings for utilization rate, there were more differences in the expenditure coefficients between the two countries, which suggests the presence of different mechanisms determining expenditures per LTC recipient in Japan and Korea.

**Table 3:** Regression Results Predicting LTC Expenditure Per Older Adult (Logged) by Country and in the Pooled Data

	**Logged LTC Expenditure Per Older Adult** ^a^		
	**Korea**	**Japan**	**Pooled**
*Demand side*			
Proportion women aged ≥65 years	3.656	2.862	3.890
	[1.870, 5.441]	[−2.793, 8.517]	[−0.048, 7.827]
Disposable income^b^	−0.045	−0.025	−0.054
	[−0.185, 0.095]	[−0.406, 0.357]	[−0.360, 0.251]
Welfare expenditure ratio^c^	0.005	1.042	0.711
	[−0.266, 0.277]	[−0.106, 2.189]	[−0.342, 1.765]
Proportion with severe eligibility levels^d^	0.516	−1.444	−0.835
	[0.085, 0.948]	[−4.003, 1.115]	[−2.455, 0.784]
Mortality rate of those aged ≥65 years	−0.641	12.003	6.336
	[−4.368, 3.087]	[−19.435, 43.441]	[−15.799, 28.472]
*Supply side*			
Physicians per 100 population	0.519	1.064	0.734
	[0.161, 0.876]	[−0.575, 2.703]	[−0.369, 1.837]
Hospital beds per 100 population	−0.117	−0.161	−0.195
	[−0.162, −0.073]	[−0.347, 0.025]	[−0.347, −0.043]
LTC institutions per 1000 population aged ≥65 years	0.052	−0.115	−0.099
	[0.010, 0.093]	[−0.175, −0.055]	[−0.151, −0.046]
Nursing workforce per 1000 population aged ≥65 years	0.000	0.001	0.000
	[−0.003, 0.002]	[−0.010, 0.011]	[−0.007, 0.007]
Proportion of LTC institutions that are government owned	−1.115	−7.003	−3.436
	[−1.871, −0.359]	[−16.703, 2.697]	[−8.684, 1.813]
*Year*			
2014	0.071	−0.010	0.002
	[0.048, 0.094]	[−0.046, 0.026]	[−0.024, 0.028]
2015	0.118	0.035	0.046
	[0.086, 0.149]	[−0.026, 0.096]	[0.003, 0.089]
Japan dummy			0.867
			[0.507, 1.227]
Constant	6.962	8.589	7.282
	[5.595, 8.330]	[5.697, 11.481]	[4.884, 9.681]

Abbreviation: LTC, long-term care. Note: Samples are weighted by the proportion of the population in the region made up of older adults. 95% confidence intervals in brackets. ^a^LTC expenditure per beneficiary with actual LTC use (in thousands of USD). ^b^Household net adjusted disposable income per capita (in tens of thousands of USD). ^c^Social welfare expenditure as a percentage of each region’s total budget. ^d^The proportion of LTCI-eligible older adults rated at the most or the second-most severe eligibility level.

 A higher proportion of older women in a region, lower disposable income, and higher welfare expenditure were related to higher LTC expenditures in both countries. In Korea, higher expenditure per beneficiary was positively associated with a higher proportion of individuals with severe eligibility levels and negatively associated with a higher mortality rate of individuals aged 65 years or over. The reverse pattern was observed in Japan.

 Regarding supply-side factors, in both countries, expenditure per beneficiary was positively associated with the number of physicians and negatively associated with the number of hospital beds and the proportion of government-owned LTC institutions. The contribution of nursing workforce per 1000 population was very low. The number of LTC institutions per 1000 population aged 65 years or over was positively related to expenditure per beneficiary in Korea but negatively related to expenditure per beneficiary in Japan.

###  Decomposition Results

####  Long-term CareUtilization Rate

Table 4 shows the decomposition results for the LTC utilization rate. The overall difference between Japan and Korea in the number of LTCI beneficiaries per older adult was 0.113 (Table 1, the first row). It was estimated that 0.101 (89.4%) of this difference can be explained by observable differences in the means of the covariates between Japan and Korea (Columns 1 and 2 in Table 4, last row), whereas only 0.012 (10.6%) of the difference can be explained by differences in the coefficients (ie, the unexplained parts; Columns 3 and 4 in Table 4, last row). This indicates that, if the supply- and demand-side attributes associated with population aging in the Korean LTC system became similar to those in the Japanese LTC system, the average LTC utilization rate in Korea would be similar to the rate in Japan.

**Table 4:** Blinder–Oaxaca Decomposition of Differences in Regression Results Predicting LTC Utilization Rate^a^ Between Korea and Japan

	**(1)**	**(2)**	**(3)**	**(4)**
	**Difference in Covariate Means**	**Contribution (%)**	**Difference in Coefficients**	**Contribution (%)**
*Demand side (aggregated)*	0.040	35.4	−0.088	−77.9
	[0.003, 0.077]		[−0.639, 0.464]	
Proportion women aged ≥65 years	0.005	4.4	−0.187	−165.5
	[−0.004, 0.013]		[−0.777, 0.402]	
Disposable income^b^	−0.006	−5.3	−0.012	−10.6
	[−0.020, 0.008]		[−0.124, 0.100]	
Welfare expenditure ratio^c^	0.024	21.2	−0.018	−15.9
	[0.005, 0.044]		[−0.063, 0.027]	
Proportion with severe eligibility levels^d^	0.012	10.6	0.103	91.2
	[−0.002, 0.027]		[−0.013, 0.218]	
Mortality rate for those aged ≥65 years	0.005	4.4	0.027	23.9
	[−0.004, 0.014]		[−0.133, 0.187]	
*Supply side (aggregated)*	0.061	54.0	0.054	47.8
	[0.037, 0.086]		[−0.042, 0.149]	
Physicians per 100 population	0.000	0.0	−0.003	−2.7
	[−0.001, 0.001]		[−0.074, 0.067]	
Hospital beds per 100 population	0.000	0.0	0.026	23.0
	[−0.003, 0.002]		[−0.026, 0.079]	
LTC institutions per 1000 population aged ≥65 years	0.057	50.4	0.031	27.4
	[0.032, 0.082]		[−0.002, 0.065]	
Nursing workforce per 1000 population aged ≥65 years	0.006	5.3	−0.005	−4.4
	[−0.002, 0.015]		[−0.092, 0.082]	
Proportion of LTC institutions that are government owned	−0.002	−1.8	0.004	3.5
	[−0.006, 0.003]		[−0.006, 0.015]	
*Year*	0.000		−0.006	−5.3
	[−0.000, 0.000]		[−0.011, −0.000]	
2014	0.000		−0.001	−0.9
	[−0.000, 0.000]		[−0.003, 0.001]	
2015	0.000		−0.005	−4.4
	[−0.000, 0.000]		[−0.008, −0.001]	
*Constant*			0.052	46.0
			[−0.453, 0.558]	
Total	0.101	89.4	0.012	10.6
	[0.055, 0.147]		[−0.033, 0.058]	

Abbreviation: LTC, long-term care. Note: Samples are weighted by the proportion of the population in the region made up of older adults. 95% confidence intervals in brackets. ^a^The number of LTCI beneficiaries with actual LTC use per adult aged 65 years or older. ^b^Household net adjusted disposable income per capita (in tens of thousands of USD). ^c^Social welfare expenditure as a percentage of each region’s total budget. ^d^The proportion of LTCI-eligible older adults rated at the most or the second-most severe eligibility level.

 Overall, the contributions of the supply-side variables (54.0%) were larger than the contributions of the demand-side factors (35.4%). A few variables substantially contributed to explaining the outcome gap through differences in the covariate means. The welfare expenditure ratio (21.2%) and the proportion of older beneficiaries with severe LTC eligibility levels (10.6%) mainly explained the contribution of demand-side factors. Regarding the supply factors, the number of LTC institutions per older 1000 population (50.4%) was the main explanation for the contribution of supply-side factors. As shown in Table 1, the supply of LTC institutions per 1000 residents was higher in Japan (6.516) than in Korea (2.633). Overall, the results indicate that, if Korea had the same mean levels of demand and supply factors, the gap between the two countries would be reduced by 89.4%.

####  Long-term CareExpenditures

Table 5 shows the decomposition results for LTC expenditures per older adult LTCI user (log-transformed). The between-country difference was 0.312 (log-transformed average; Table 1), which indicates that the average LTC expenditures per service recipient were approximately 40% higher in Japan than in Korea. The decomposition results in Table 5 show that the gap between the two countries in LTC expenditures per recipient would be even larger and would reach 0.867 (= 0.312 + 0.555, or −177.9%, Column 2, last row) if the differences in the observed distributions of supply and demand disappeared.

**Table 5:** Blinder–Oaxaca Decomposition of Differences in Regression Results Predicting LTC Expenditure Per Beneficiary (Logged)^a^ between Korea and Japan

	**(1)**	**(2)**	**(3)**	**(4)**
	**Difference in Means**	**Contribution (%)**	**Difference in Coefficients**	**Contribution (%)**
Demand side (aggregated)	−0.198	−63.5	−0.266	−85.3
	[−0.437, 0.041]		[−3.625, 3.092]	
Proportion women aged ≥65 years	−0.055	−17.6	−0.187	−144.9
	[−0.115, 0.005]		[−0.777, 0.402]	
Disposable income^b^	−0.015	−4.8	−0.012	12.8
	[−0.096, 0.066]		[−0.124, 0.100]	
Welfare expenditure ratio^c^	−0.097	−31.1	−0.018	78.8
	[−0.236, 0.041]		[−0.063, 0.027]	
Proportion with severe eligibility levels^d^	−0.049	−15.7	0.103	−170.5
	[−0.141, 0.042]		[−0.013, 0.218]	
Mortality rate for those aged ≥65 years	0.019	6.1	0.027	138.1
	[−0.045, 0.084]		[−0.133, 0.187]	
Supply side (aggregated)	−0.358	−114.7	−0.438	−140.4
	[−0.550, −0.166]		[−1.035, 0.160]	
Physicians per 100 population	0.001	0.3	−0.003	42.3
	[−0.020, 0.022]		[−0.074, 0.067]	
Hospital beds per 100 population	0.003	1.0	0.026	−20.2
	[−0.046, 0.051]		[−0.026, 0.079]	
LTC institutions per 1000 population aged ≥65 years	−0.383	−122.8	0.031	−160.6
	[−0.580, −0.187]		[−0.002, 0.065]	
Nursing workforce per 1000 population aged ≥65 years	0.000	0.0	−0.005	11.2
	[−0.052, 0.052]		[−0.092, 0.082]	
Proportion of LTC institutions that are government owned	0.022	7.1	0.004	−12.8
	[−0.016, 0.060]		[−0.006, 0.015]	
Year	0.000	0.0	−0.055	−17.6
	[−0.000, 0.001]		[−0.091, −0.020]	
2014	0.000	0.0	−0.001	−8.7
	[0.000, 0.000]		[−0.003, 0.001]	
2015	0.000	0.0	−0.005	−9.0
	[0.000, 0.001]	0.0	[−0.008, −0.001]	
Constant			1.626	521.2
			[−1.559, 4.811]	
Total	−0.555	−177.9	0.867	277.9
	[−0.878, −0.232]		[0.525, 1.209]	

Abbreviation: LTC, long-term care. Note: Samples are weighted by the proportion of the population in the region made up of older adults. 95% confidence intervals in brackets. ^a^LTC expenditure per beneficiary with actual LTC use (in thousands of USD). ^b^Household net adjusted disposable income per capita (in tens of thousands of USD). ^c^Social welfare expenditure as a percentage of each region’s total budget. ^d^The proportion of LTCI-eligible older adults rated at the most or the second-most severe eligibility level.

 The differences in coefficients, in contrast, contributed positively to the between-country gap in LTC expenditure. This contribution (Column 4, last row) was large enough to offset the contribution of the differences in the covariate means (0.867, or 277.9%). The contrasting contributions by the coefficients were complex; the aggregated contribution of demand (−85.3%) and supply factors (−140.4%) was offset by the constant (521.2%). This finding suggests that, unlike the case for the LTC utilization rate, the between-country gap in LTC expenditures per beneficiary could more likely be explained by differences in unique institutional factors than by differences in the means of supply and demand attributes. The results further indicate that institutional factors in Japan were associated with cost containment, for the levels of supply and demand factors in Japan, in contrast to the Korean institutional situation.

## Discussion

 The results of this decomposition analysis comparing LTC social insurance schemes in Korea and Japan showed that the utilization rate of formal LTC services is likely determined by the distribution of factors such as demographic and functional conditions of the target population, the financial capacity of local governments, and supply responses in the region, regardless of institutional differences between the two countries. In both countries, a high proportion of women aged 65 years or over, higher disposable income, and high spending on welfare programs were related to lower utilization rates, indicating that provisional capacity of informal care and access to substitutional welfare services may reduce the likelihood of a household to use formal care services. This finding also suggests that the two countries share common mechanisms that determine consumer choice behaviors related to formal service utilization under public insurance schemes.

 One previous study in Europe found that the utilization rate of LTC formal services of any kind was only 10% in the Netherlands and 3.6% in Germany,^5,6^ much lower rates than those for Japan and Korea. Because demand for formal LTC in Japan and Korea developed in the contexts of higher population aging rates compared with European countries and social pressures for informal care provision encouraged by Confucianism culture, consumer decisions to use formal care to complement informal care are likely to be more prevalent in these settings.^7,10^

 In contrast, expenditures per beneficiary, or the intensity of service utilization, appeared to be determined more by unexplained country-specific institutional factors that are presumably related to the governance structure and choice of policy targets for LTC coverage. In both countries, there were very few strong predictors of expenditure per beneficiary for both demand and supply factors. This finding suggests that unexplained institutional factors such as policy generosity and operation design may be more influential in expenditure control than demographic change and subsequent demand increase for LTC services owing to population aging.

 The Japanese LTC system was designed to complement informal care at middle-income households that is mainly provided by family caregivers.^8^ In contrast, the Korean LTC system was generous to provide formal institutionalized care to people with high care needs to relieve family caregivers of care and economic burdens.^9^ These different choices regarding policy targets may have caused the observed differences between the two countries in the associations of the population proportions both of older women and of those with severe care needs with the amount of expenditures per beneficiary. Since the LTC utilization rate, the less controllable trend of population aging, is determined more by demographic and socioeconomic conditions, the results of the current study suggest that policy-makers need to make flexible strategic choices over time about LTC institutional schemes to maintain financial sustainability while meeting changing demands caused by population aging.

 Although the discussion of the financial scheme for long-term service provision has been the central theme of international discussion around LTC policy,^25^ the resource allocation after the implementation of LTC system has been relatively ignored.^12^ A previous cross-country analysis among OECD countries found a common trend where resource allocation for community-based services prioritized coverage (population portion eligible for service use) over resource intensity (expenditure per capita), while the reverse was true for residential care.^11^ However, the difference in resource allocation between coverage and intensity and its influential factors are understudied mainly because of a lack of comparative data across countries.^12^ We chose the data of years 2013-2015 in consideration of the comparability of LTCI systems and data in Japan and Korea. A careful definition of service coverage and data comparability are fundamental requisites for international comparative analysis of LTC expenditure.^13,26^ By careful data preparation, the current study specifically aimed to fill a knowledge gap by focusing on the cross-country variation in coverage and intensity.

 The difference in the LTC governance structure between Korea (centralized) and Japan (decentralized) may also warrant discussion in relation to policy implications. A similar comparison was conducted between Italy and Spain, where, to differing degrees, decentralized governance structures place the main responsibility for healthcare policy on regional governments.^27^ The results from this previous study showed that decentralization per se may not be the main source of regional variance; rather, the quality and efficiency of the government structure and system design seem to be the main determinants of these differences. This argument can be extended to the cases of Korea and Japan.

 A strength of the present study is the comparison of LTCI systems in two countries with similar political and cultural backgrounds, which permitted a closer focus on the impact of institutional differences on service coverage and intensity. Our finding of a systemic between-country difference only in expenditure per beneficiary and not in the likelihood of formal service use may not be well explained solely by differences in preference and culture between Korea and Japan.

 The current study did not evaluate the financial scheme for LTC provision and political drivers for LTC system introduction because these factors are highly specific to the existing healthcare financing scheme, culture, and politico-historical path of each country, which precludes comparative analysis.^13^ Furthermore, although private funding for LTC provision has proven significance,^28^ the current study did not include private funding because cross-country comparative data on this issue are very scarce.^13^ Our study presumed that LTC provision was covered under public funding through the social insurance and tax subsidy scheme. Recent discussion on multi-pillar financing composed of public funding with a pay-as-you-go scheme, tax-based means-tested payment, asset saving, and voluntary private insurance sought a sustainable and feasible scheme to overcome the limitation of the pay-as-you-go system under an increasing proportion of dependents.^25^ However, the public financing scheme is expected to cover basic LTC service provision,^25^ and the current study provides information for resource allocation under a public insurance scheme for policymakers seeking to create an efficient and manageable LTC policy.

 There were several additional study limitations. First, we used prefectures in Japan and provinces in Korea as regional units of analysis; however, the population size and functional roles of these units within the overall system of each country may not necessarily be comparable. Additionally, the use of aggregated data may have led to ecological fallacy in interpreting the results. Future individual-based comparisons are warranted if data availability issues can be resolved. Second, although we relied on a previous analytic scheme of predisposing, enabling, and need factors, we cannot rule out the possibility of misspecification of relevant analytic variables owing to limited availability of comparable aggregated data items at regional levels. Third, we basically relied on linear regression models, and there may be violations of regression assumptions. We conducted regression diagnoses to examine the model fit. Although we did not observe serious heteroscedasticity, we did identify a small multicollinearity problem (ie, the average variance inflation factor of the pooled models was 2.95). In Japan, a higher proportion of severe eligibility levels unexpectedly showed a negative association with expenditure per beneficiary due to regression outliers with a high proportion of severe eligibility levels and low expenditure in some urban prefectures (data not shown). Again, future studies are needed that use larger individual-based administrative claims data combined with institutional-level statistics, and multilevel regression analyses, to specify the function of LTC utilization rate and expenditure per beneficiary. Fourth, our analysis may not fully adjust for the difference in age proportions between the two countries. We limited our data to the population aged 65 years and over to maintain data comparability in accordance with previous studies.^11,29^ However, the proportion of the population aged 80 years and over was markedly different between the two countries (3% in Korea and 8% in Japan in 2015).^30^ Unfortunately, outcome data regarding utilization rates and expenditure per capita in those aged 80 years and older are not available for our analyses. Future studies are needed to determine whether the greater proportion of people older than 80 years could further explain between-country differences in population coverage and expenditure per recipient.

## Conclusion

 The current findings suggest that the LTC utilization rate is largely determined by the demographic characteristics and functional status of older adults and by the supply response, and policy responses to these challenges are necessarily reactive rather than proactive. Instead, variations in expenditures are more affected by institutional factors such as insurance policy design and governance structure, so should be the main focus of policy design. The results suggest that policy-makers should strategically consider the institutional design of LTC schemes for cost control to efficiently meet increasing demands caused by demographic change.

## Acknowledgments

 The authors acknowledge Ms. Mutsumi Tokunaga and Dr. Seyune Lee for their assistance with the data preparation.

## Ethical issues

 Ethical approval for this study was obtained in Korea (Seoul National University Internal Review Board No. E1807/003-003). In Japan, the requirement for ethical approval was waived by the University of Tokyo Graduate School of Medicine because the study was a secondary analysis of publicly available data or anonymized data with official use approval for research purposes from governmental agencies.

## Competing interests

 Authors declare that they have no competing interests.

## Authors’ contributions

 HK developed the study idea, drafted the manuscript, and contributed to the interpretation of the results and the critical revision of the manuscript. NHY conducted the data collection and analysis and contributed to the interpretation of the results and the writing. YH conducted the data analyses and data interpretation and contributed to the writing. HH developed the study design, conducted the data collection and analysis, and contributed to the interpretation of the results and the critical revision of the manuscript.

## Disclaimer

 Opinions and views included in this manuscript are of the authors’ own, and do not constitute any view/positions of affiliated institutes or of owners of original data.

## Funding

 HK were supported by an AXA Award from the AXA Research Fund, Paris, France (900-2017006), the Humboldt Research Fellowship from the Alexander von Humboldt Foundation, Bonn, Germany, and the National Research Foundation of Korea (NO.419 999 0514025); and HH was supported by the Ministry of Education, Culture, Sports, Science and Technology (18H04070 Grant in Aids for Scientific Research [A]). The funding sources had no involvement in the design or conduct of the study or in the data collection, data management, data analysis and interpretation, preparation, or review and approval of the manuscript.

## Supplementary files

Supplementary file 1. Detailed Description of Public Long-term Care Insurance Systems in Korea and Japan.

Supplementary file 2. Detailed Description of Analytic Methods of Oaxaca–Blinder Decomposition.
