# Supplementary material for: Explaining Variations in Long-term Care Use and Expenditures Under the Public Long-term Care Insurance Systems: A Case Study Comparison of Korea and Japan
Source: Int J Health Policy Manag. 2022 Nov 19;12:6640. doi: 10.34172/ijhpm.2022.6640 (PMC10125049; doi:10.34172/ijhpm.2022.6640)
Supplement: Supplementary file 1 — Detailed Description of Public Long-term Care Insurance Systems in Korea and Japan. [file ijhpm-12-6640-s001.pdf]

Article title: Explaining Variations in Long-term Care Use and Expenditures Under the Public Long-term Care Insurance Systems: A Case Study Comparison of Korea and Japan

Journal name: International Journal of Health Policy and Management (IJHPM)

Authors' information: Hongsoo Kim[1,2], Nan-He Yoon[3], Youjin Hahn[4], Hideki Hashimoto[5]*

[1]Graduate School of Public Health Department of Public Health Sciences, Institute of Health and Environment & Institute of Aging, Seoul National University, Seoul, Republic of Korea.

[2]SOCIUM - Research Center on Inequality and Social Policy, University of Bremen, Bremen, Germany.

[3]Division of Social Welfare and Health Administration, Wonkwang University, Iksan, Republic of Korea.

[4]School of Economics, Yonsei University, Seoul, Republic of Korea.

[5]Department of Health and Social Behavior, The University of Tokyo School of Public Health, Tokyo, Japan.

(*Corresponding author: hidehashim@m.u-tokyo.ac.jp)

Supplementary file 1. Detailed Description of Public Long-term care Insurance Systems in Korea and Japan

Although there are several common features in the basic design of the LTCI programs in Japan and Korea, there are also unique aspects in their financing and delivery of LTC.[31] In this section, we provide a brief overview of the governance/financing, provision bodies, and service benefits of the two countries' public LTCI systems because these characteristics may be relevant in explaining regional and cross-country variation in LTC utilization.

Japan established its social insurance-based system for the formal provision of LTC in 2000, following Germany's LTCI system. In Japan, LTCI is mandatory, with individuals aged 40 years or older paying a premium. The system provides formal LTC services for those aged 65 years or older and those with designated disability conditions who are approved as eligible following nationally standardized eligibility criteria. The public

LTCI covers personal care at home, community-based respite care, and institutionalized personal care with a fixed-rate copayment.[32]

Korea adopted a similar scheme when it implemented its LTCI system in 2008; however, in the Korean system, premium contributions are paid by all households to allow wider intergenerational transfer. The two countries share cultural norms regarding seniority and the family, which normatively oblige private families to serve as primary caregivers for frail older adult relatives.[33]

When Japan implemented its public LTCI system in 2000, the percentage of the population aged 65 years or older had already reached 17.3%, whereas this percentage in Korea was approximately 10% as of 2008.[32.33] The Japanese LTCI was expected to meet a wide range of demands for LTC, including mild care needs in middle-income households in which the burden of informal caregiving had become overwhelming but purchasing formal care out-of-pocket was not affordable. Consequently, LTC provision in Japan shifted toward home- and community-based care. In contrast, the Korean LTCI focused on giving high-need groups easier access to institutional care as a prioritized choice of service.

Another difference between the two countries' systems is the insurer scheme.[31] Japan uses a multipayer system with more than 2500 local municipal government insurers, whereas Korea has adopted a single-payer system (the National Health Insurance Service; NHIS). In each country, the development of the LTC insurer scheme was based on a pre-existing public health insurer scheme. In Japan, LTCI beneficiaries pay different premium rates across local regions (ranging from JPY 7800 to JPY 34,500, or USD 71–314 equivalent per month in 2019). In Korea, in contrast, beneficiaries pay a standardized universal premium rate. Both countries offer nationally standardized packages of benefits and payment schemes.

The financing schemes are also distinctive in the two countries.[31-33] The Japanese decentralized system obtains 50% of its funding from local premium revenue, 25% from tax transfers from the central government, and 25% from tax transfers from local (prefecture and municipal) governments. The central government tax transfers were included in the scheme to compensate for regional differences in premium rates and in the financial capacity of local governments. The Korean system is 80% funded by premium revenue collected by the single government payer; the remaining 20% is

covered by tax transfers from the central government for the NHIS and from local governments to allow for premium exemptions for low-income households.

Both countries' LTCI systems rely heavily on the private sector for service delivery, which is paid on a fee-for-service basis under nationally standardized fee schedules. In Japan, most of the relevant private sector actors are non-profit organizations, whereas providers with private ownership are more dominant in Korea.[32,33] For-profit providers are allowed to provide homecare in Japan, but they are strictly prohibited from doing so in Korea, although providers with private ownership can behave like for-profit providers. Finally, providers in Japan are required to obtain certification from the prefectural government, which monitors and controls the number of providers according to the need in each region.[32] In contrast, the Korean system has no such certificate-of-need policy to officially regulate market entry, which can lead to oversupply and excessive competition in some regions.[33]

After being approved as eligible, beneficiaries in Japan are allowed to choose the service types they receive within their monthly limit, with help from certified service coordinators called "care managers." The general copayment rate is 10% regardless of the service type in Japan, and individuals with higher incomes are required to pay 20%. Eligibility approval and free service choice have similarly been adopted in Korea, although the Korean LTCI system has not implemented a care coordinator program. The copayment rate is approximately 20% for institutional-care users and 15% for home- and community-based service users in Korea.

Japan has a shortage of care service personnel and an institutional-care capacity that is unable to fully meet the rapidly increasing demand.[32] In contrast, there is an oversupply of these resources in Korea, but shortages in the specialty work force (e.g., home-visit nurses and doctors) and infrastructure limitations are considered serious barriers to extending home- and community-based health services under the public LTCI system.[33]

References for Appendix 1

31. Campbell J, Ikegami N, Kwon S. Policy learning and cross-national diffusion in social long-term care insurance: Germany, Japan, and the Republic of Korea. *Int Soc Secur Rev.* 2009;62(4):63-80. doi:10.1111/j.1468-246X.2009.01346.x

32. Tamiya N, Noguchi H, Nishi A, et al. Population ageing and wellbeing: lessons from Japan's long-term care insurance policy. *Lancet.* 2011;378(9797):1183-1192. doi:10.1016/S0140-6736(11)61176-8
33. Kim H, Kwon S. A decade of public long-term care insurance in South Korea: policy lessons for aging countries. *Health Policy.* 2021;125(1):22-26. doi:10.1016/j.healthpol.2020.11.003
