# Supplementary material for: Explaining Variations in Long-term Care Use and Expenditures Under the Public Long-term Care Insurance Systems: A Case Study Comparison of Korea and Japan
Source: Int J Health Policy Manag. 2022 Nov 19;12:6640. doi: 10.34172/ijhpm.2022.6640 (PMC10125049; doi:10.34172/ijhpm.2022.6640)
Supplement: Supplementary file 2 — Detailed Description of Analytic Methods of Oaxaca–Blinder Decomposition. [file ijhpm-12-6640-s002.pdf]

Article title: Explaining Variations in Long-term Care Use and Expenditures Under the Public Long-term Care Insurance Systems: A Case Study Comparison of Korea and Japan

Journal name: International Journal of Health Policy and Management (IJHPM)

Authors' information: Hongsoo Kim[1,2], Nan-He Yoon[3], Youjin Hahn[4], Hideki Hashimoto[5]*

[1]Graduate School of Public Health Department of Public Health Sciences, Institute of Health and Environment & Institute of Aging, Seoul National University, Seoul, Republic of Korea.

[2]SOCIUM - Research Center on Inequality and Social Policy, University of Bremen, Bremen, Germany.

[3]Division of Social Welfare and Health Administration, Wonkwang University, Iksan, Republic of Korea.

[4]School of Economics, Yonsei University, Seoul, Republic of Korea.

[5]Department of Health and Social Behavior, The University of Tokyo School of Public Health, Tokyo, Japan.

(*Corresponding author: hidehashim@m.u-tokyo.ac.jp)

Supplementary file 2. Detailed Description of Analytic Methods of Oaxaca–Blinder Decomposition

First, we used linear regression models to examine factors explaining the LTC utilization rate and LTC expenditures in each country.

More specifically, we estimated the following model for each country:

$$Y_{it} = \alpha + \gamma_1 Predisposing_{it} + \gamma_2 Enabling_{it} + \gamma_3 Need_{it} + \gamma_4 Supply_{it} + \delta_t + \varepsilon_{it}, \quad (1)$$

where Y_{it} is the outcome variable for region *i* and year *t*, and δ_t represents a set of year fixed effects. In the model, *Demand* (*Predisposing*, *Enabling*, and *Need* factors) and *Supply* are vector variables, as described in the previous section. We weighted the sample by the proportion of the population in each region made up of older adults and estimated the model using weighted least squares. To account for multiple observations per region/prefecture over the years, sandwich estimation of standard errors clustered by region/prefecture was used.

Next, we decomposed the outcome gap between Korea and Japan using the decomposition technique proposed by Oaxaca[34] and Blinder[35] to determine whether a between-country gap is caused by differences in the means of the covariates ($E(X_{JP}) - E(X_{KR})$) or by differences in the coefficients. The between-country gap can be decomposed in the following way:

$$E(Y_{JP}) - E(Y_{KR}) = \hat{\beta}^{*\prime}\left[E(X_{JP}) - E(X_{KR})\right] + (\hat{\beta}_{JP} - \hat{\beta}^{*})' E(X_{JP}) + (\hat{\beta}^{*} - \hat{\beta}_{KR})' E(X_{KR}), \quad (2)$$

where the first term represents the portion of the difference explained by the means of the covariates, reflecting the effect of between-country differences in the distributions of the observed explanatory variables. The second and third terms together represent the portion of the difference that is caused by the coefficients and indicate the difference not explained by the observed variables, which presumably reflects the difference attributable to unmeasured institutional differences between the two countries' systems.

The portion of the difference caused by differences in the means of the covariates for each variable was calculated as $\hat{\beta}^{*\prime}\left[E(X_{JP}) - E(X_{KR})\right]$. The coefficient $\hat{\beta}^{*}$ has been estimated in several ways in the literature. Following Neumark,[36] we used the coefficients for $\hat{\beta}^{*}$ obtained by regressing Y on the covariates in the pooled sample, including a

country indicator as a control variable. The portion of the difference caused by coefficient differences for each variable was calculated as

$(\hat{\beta}_{JP} - \hat{\beta}^{*})'E(X_{JP}) \; + (\hat{\beta}^{*} - \hat{\beta}_{KR})'E(X_{KR})$.

References for Appendix 2

31. Oaxaca R. Male-female wage differentials in urban labor markets. *Int Econ Rev.* 1973;14(3):693-709. doi:10.2307/2525981

32. Blinder A. Wage discrimination: reduced form and structural estimates. *J Human Resources.* 1973;8(4):436-455. doi:doi:10.2307/144855

33. Neumark D. Employers’ discriminatory behavior and the estimation of wage discrimination. *J Human Resources.* 1988;23(3):279-295. doi:10.2307/145830
